# Numerical Simulation of the Smoke Recirculation Behavior in Street Canyons with Different Aspect Ratios and Cross-Wind Conditions

**DOI:** 10.3390/ijerph19127056

**Published:** 2022-06-09

**Authors:** Yanqing Xiang, Kaihua Lu, Jie Wang, Yanming Ding, Shaohua Mao

**Affiliations:** 1 Faculty of Engineering, China University of Geosciences Wuhan, Lumo Road 388, Wuhan 430074, China; 1202010524@cug.edu.cn (Y.X.); dingym@cug.edu.cn (Y.D.); maoshaohua@cug.edu.cn (S.M.); 2 School of Resource and Environmental Engineering, Wuhan University of Science and Technology, Heping Avenue 947, Wuhan 430081, China; wangjie87@wust.edu.cn

**Keywords:** smoke dispersion, street canyon, aspect ratio, critical recirculation velocity, cross-wind

## Abstract

This study investigated smoke dispersion inside a street canyon in a series of numerical simulations. The building height and street width as well as the cross-wind velocity were changed during the simulation, and the smoke recirculation behavior inside the canyon is presented and discussed. The results show that the smoke recirculation behavior could be distinguished into two different stages, i.e., the “fully recirculation stage” and “semi recirculation stage”, which is strongly determined by the canyon aspect ratio (the building height divided by street width). It was found that the critical wind velocity at which the smoke recirculation would take place was almost constant for an ideal street canyon with an aspect ratio of 1; however, this velocity was decreased with increasing building height or decreasing street width, indicating a much more dangerous circumstance when the aspect ratio is greater. Finally, a new piecewise function is proposed for the critical smoke recirculation velocity for all cases, which can provide some theoretical basis for building designs and emergency rescue for human beings inside the street canyon.

## 1. Introduction

Street canyons, consisting of one street area with tall buildings standing on both sides of the street [1], have been widely applied in modern cities to keep pace with the rapid development of urban constructions. There are many commercial and residential buildings inside the canyon, and, regarding potential environmental issues to public safety and human health, the diffusion model of pollutants in urban street canyons has attracted more and more attention in recent decades [2,3,4,5]. Normally, the aspect ratio of street canyons (*n* = H/W, where H and W are the building height and street width, respectively) is a key factor determining the diffusion of pollutants [6,7]; pollutants can be dispersed into the surroundings more easily in a street canyon with a lower aspect ratio [8]. The flow pattern in the street canyon when the incoming ambient wind is perpendicular to the building direction was analyzed by Oka [9], who concluded that the flow field fell into three different regimes according to the variations in the aspect ratio of the street canyon, i.e., isolated roughness flow (with *n* < 0.3), wake interference flow (with 0.3 < *n* < 0.7) and skimming flow (with *n* > 0.7). A change in the above flow pattern would influence the entrainment of ambient air to the pollutants inside the canyon, and hence, the diffusion behavior of pollutants could be different [10,11].

Since then, weak buoyancy-driven flow behavior in different kinds of street canyons has been addressed all around the world, and the most representative factors are solar radiation, ground heating, etc. For instance, Kim [12] studied variations in pollutant diffusion within a ground-heated street canyon by changing the aspect ratio from 0.6 to 3.6. Wang [13] found that the thermal buoyancy generated by solar radiation heating on both the ground and buildings could significantly affect the flow field pattern in the street canyon. Furthermore, the natural convection field in urban street canyons under the condition of uniform heating by solar radiation on the street canyon ground and buildings on both sides was investigated [8,14,15,16,17,18]. More recently, the flow pattern and temperature field in the street canyon for different heating conditions were acquired by Lin [19] through the particle image velocimetry method (PIV), indicating that the presence of thermal buoyancy can significantly enhance the turbulence in the street canyon, resulting in a more complex flow field in the street canyon.

It should be noted that all of the above studies are based on the study of the street canyon under no thermal buoyancy or weak thermal buoyancy conditions; however, as we know, the street canyon is always a densely populated area with a large number of fire causative factors. When a fire occurs in the street canyon (e.g., car collisions), the strong buoyancy caused by fire can make the flow dispersion completely different. In the meantime, strong buoyancy-driven flows will also be affected when the ambient wind is stronger, resulting in the most representative smoke recirculation behavior inside the canyon. In recent years, some scholars started to investigate the smoke recirculation behavior under different street canyon configurations. The important concept of “critical recirculation velocity” was initially proposed by Hu [20]; namely, when the cross-wind velocity is greater than this critical level, fire smoke will be recirculated back inside the canyon, imposing much more serious fire and smoke hazards for evacuation and emergency rescue. The critical recirculation velocity for different fire locations and heat release rates was further obtained by Hu [20,21], Pesic [22,23,24] and Wang [25], and then a dimensionless factor called the Froude number (denoted as Fr) was introduced to reflect the competition of inertia force (due to ambient wind) versus thermal buoyancy (due to fire). Zhang [26] studied an urban street canyon formed by wedge roof buildings and found that the flow pattern of pollutant plume dispersion inside the street canyon with increasing wind speed for different roof inclination angles could be divided into three regimes.

However, we have noticed that most of the previous studies are concerned with fires in an ideal street canyon with a height–width aspect ratio of 1 [27,28,29], but in real life, we can see many different kinds of street canyons. Considering the different flow patterns due to various aspect ratio conditions, when a fire occurs in the street canyon, the aspect ratio effect on the smoke dispersion could be another issue to be addressed. The aspect ratio effect on the critical wind velocity and CO concentration was discussed by Zhang [30] and Hu [31]. It was found that the critical wind velocity does not fall into a simple linear correlation; however, the building height did not surpass 24 m in their models, which might not be enough. Physically, if the building is high enough, the smoke may not completely sink to the ground as a result of mechanical balance on the facade wall of a leeward building, but this has not been addressed in previous investigations.

For this purpose, this paper aims to further investigate the aspect ratio effect on smoke dispersion in a street canyon. Applying the well-known Fire Dynamic Simulator software (FDS), a series of street canyon models with building heights ranging from 9–36 m and street widths of 12–24 m were set up to cover different canyon aspect ratios ranging from 0.38–3. Keeping the heat release rate (HRR) of fire constant at 5 MW according to normal combustion due to vehicle collision, the flow patterns of smoke dispersion were compared and analyzed. Finally, the relationship between critical recirculation velocity and the canyon aspect ratio was determined and discussed. This study could provide some theoretical basis for building designs and the emergency rescue of human beings inside the street canyon.

## 2. Modeling

### 2.1. Physical Model Configuration

A 1:1 street canyon model was set up by using the software of Fire Dynamic Simulator (FDS, version 6) developed by the National Institutes of Standards and Technology (NIST), which is a well-known CFD-based software in fire engineering. In order to minimize the calculation duration, the Large-Eddy Simulation (LES) method is predefined in FDS modeling. The governing equations for LES simulation, which consist of conservation of mass, momentum and the transport of sensible enthalpy, can be found in McGrattan et al. [32]. Previous studies [20,21] have verified the accuracy of FDS model predictions in terms of wind velocity fields, gas diffusion characteristics and plume parameters under wind conditions, respectively.

**Figure 1:**
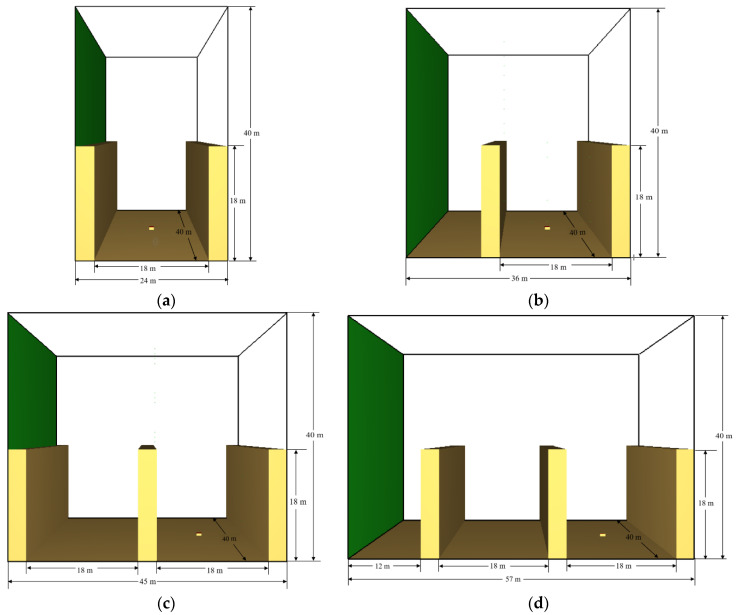
Two categories of wind–building configuration: (**a**) single-street canyon model with wind inlet adhered to the building; (**b**) single-street canyon model but with a small gap between the wind inlet and the building; (**c**) multiple-street canyon model with wind inlet adhered to the building; (**d**) multiple-street canyon model but with a small gap between the wind inlet and the building.

In this study, we thought that the association between the wind and building could be complex. In general, there are two categories of wind–building configuration: in category (a), the wind inlet adheres to the left side of the first building, and in category (b), a small gap occurs between the wind inlet and the left side of the first building (see Figure 1 below, containing a single-street canyon model and a multiple-street canyon model).

**Figure 2:**
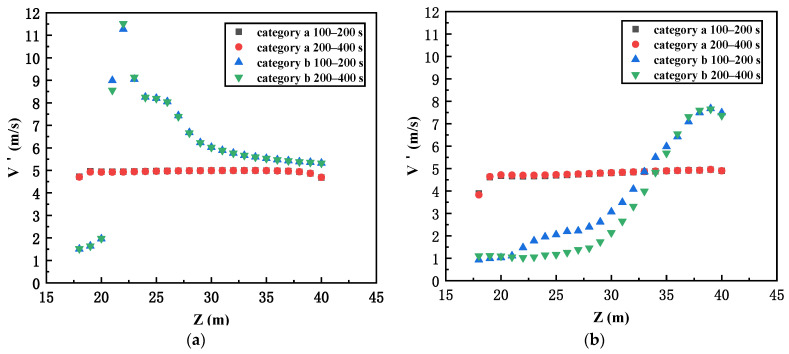
The vertical wind profile at the entrance of the last street canyon: (**a**) single-street canyon model; (**b**) multiple-street canyon model.

Thus, it should be noted that the two-category configuration could cause the results to be completely different. As seen in Figure 2, for the category (a) cases, except for a short distance above the building top, the wind velocity profile is relatively uniform at the entrance of the last street canyon. This would not have a strong impact on the smoke recirculation inside the street canyon. However, for category (b) cases, the wind velocity profile is completely different at the entrance of the last street canyon and is not stable (e.g., in Figure 2a, the maximum wind velocity can be almost 11 m/s and then finally reduced, but the maximum wind velocity can be lower in Figure 2b).

**Figure 3:**
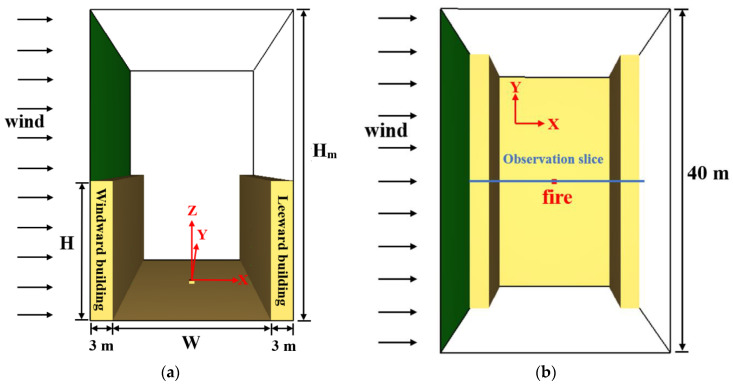
Model configuration of the street canyon with different aspect ratios: (**a**) 3D model in FDS; (**b**) top view (H—building height; W—separation distance; and H_m_—height of the calculation domain).

To simplify the model configuration and avoid wasting calculation resources, we set up a single-street canyon model, as shown in Figure 3, with two parallel buildings (40 m in length and 3 m in width) located on both sides of a street. The building height (H) and separation distance (W) are changeable to represent different aspect ratio conditions (*n* = H/W) of the street canyon. The height of the building ranges from 9 m to 36 m, whereas the building separation distance is set to 12 m, 15 m, 18 m, 21 m or 24 m. It should be noted that the above configuration matches well with a typical street canyon in a city (e.g., 3–6 traffic lanes inside the canyon and 3–12 levels for the building alongside the street, while the aspect ratio *n* = H/W also matches well with practical building configurations, which is strongly associated with sunlight, ventilation and population requirements). In order to limit the errors caused by modeling, a calculation domain of 40 m in length and 24 m in width was created, the height of which (H_m_) increases with the height of the building (H), due to the effect of areas of insufficient height on the airflow over the roof of the building. A fire source fueled by n-heptane was set in the center of the street canyon with a constant heat release rate (HRR) of 5 MW, reflecting a fire scenario due to small car collisions inside the canyon.

Then, a uniform inlet velocity boundary condition was set on the left side of the simulation domain as representative of the ambient cross-wind. The configuration abides by some previous works by Hu [20,21,31], Zhang [26,30] and Pesic [23], but the wind profile was not considered in these works. The wind direction was perpendicular to the building, but the velocity of the cross-wind was altered step by step in the simulation to search for the critical smoke recirculation velocity of the street canyon, which was determined visually from the smoke dispersion throughout the simulation time period. The top and the other three sides of the domain were set as free boundary conditions. Related studies [33] have shown that for a street canyon with W/H = 1, FDS still predicts the flow field measurements from wind tunnel experiments, and predictions are in good agreement with uniform inflow boundary conditions where no turbulence intensity is specified. It was also shown [34] that in the skimming flow regime for such a square canyon, the level of turbulence within the canyon is not very sensitive to external turbulent conditions because the shear layer at the interface acts as a filter for the incoming turbulent structure. In order to have a better view of the smoke dispersion behavior and flow pattern, an observation slice was employed through the centerline in the Y direction of the street canyon. The simulation time was 400 s when the flow field and pollutant distribution in the street canyon reached stability. Initial conditions were set to an ambient temperature of 20.0 °C, ambient pressure of 1.0 atm, relative humidity of 40.0%, ambient oxygen mass fraction of 23% and ground level of 0.0 m. All the simulation conditions are listed in Table 1.

### 2.2. Grid Sensitivity

**Figure 4:**
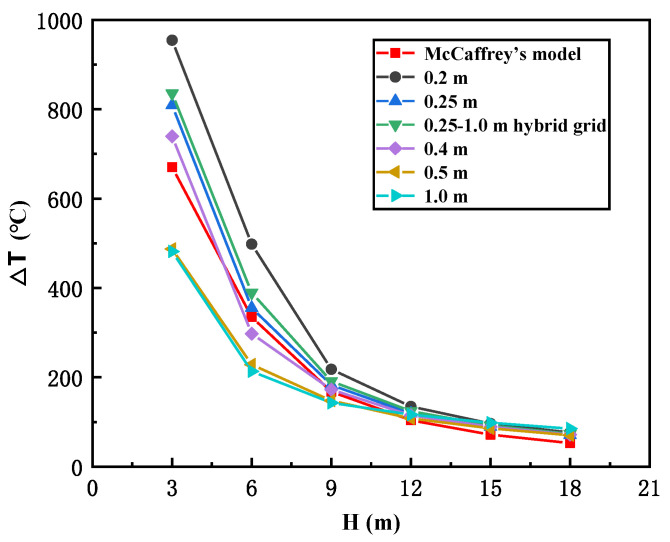
Comparison of different cubic mesh sizes with McCaffrey’s model.

Before the simulation, normally, we need to check the grid size to see if it is appropriate to accomplish the simulation. According to the FDS user’s guide [35], the validation study sponsored by the U.S. Nuclear Regulatory Commission [36] and some relevant research works [37], when the grid size *δx* is between D*/16 and D*/4, the simulation can be well established, in which D* is the characteristic diameter relative to HRR of the fire source:

(1) $$D^{*}={\left(\frac{\dot{Q}}{\rho_{\infty }C_{p}T_{\infty }\sqrt{g}}\right)}^{2/5}$$

where $\dot{Q}$ is HRR; $\rho_{\infty }$ is the ambient air density; *C*_p_ is the specific heat of air; *T_∞_* is the ambient air temperature; and *g* is the acceleration of gravity. On this basis, the mesh size is preferred to be 0.11–0.46 m. Here, taking the fire scenario of H = W = 18 m with no cross-wind as a reference, a series of cubic mesh dimensions, i.e., 0.2 m, 0.25 m, 0.4 m, 0.5 m and 1.0 m, were selected for comparison with McCaffrey’s model, which is the most representative one regarding the temperature profile of fire plumes. Based on the thermocouple results at 3 m, 6 m, 9 m, 12 m, 15 m and 18 m above the fire source, it is seen in Figure 4 that, when the grid size is 0.25 m, the simulation results are more similar to McCaffrey’s model, but the calculation duration would be still very extensive. Then, to reduce calculation resources to the greatest extent, a 0.25–1.0 m hybrid grid size was applied in this study; i.e., for the near-fire region with −10 m ≤ Y ≤ 10 m, the grid size was 0.25 m, but for other regions somewhat farther from the fire source (−20 m < Y < −10 m, 10 m < Y < 20 m), the grid size was changed to 1.0 m. The results show that the 0.25–1.0 m hybrid grid size is similar to the 0.25 m cubic grid, so it can be applied to accomplish the simulation, while the calculation duration can also be significantly shortened.

## 3. Results and Discussion

### 3.1. Smoke Recirculation Behavior in Street Canyons

**Figure 5:**
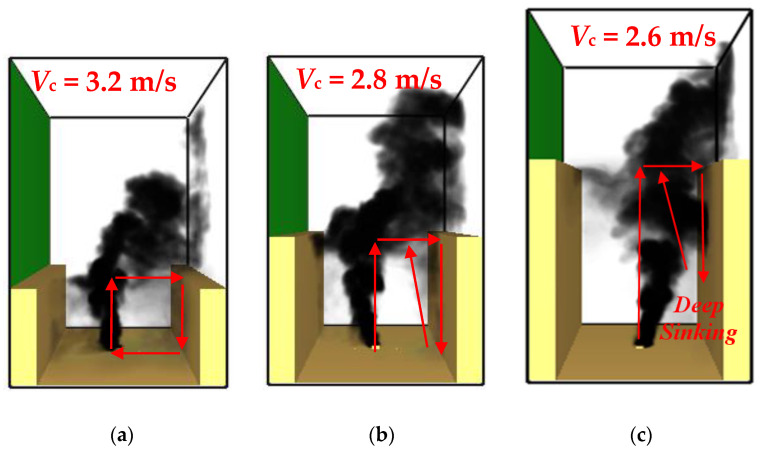
The critical velocity of smoke for different building heights at 400 s: (**a**) H = 12 m, *V_c_* = 3.2 m/s; (**b**) H = 18 m, *V_c_* = 2.8 m/s; and (**c**) H = 24 m, *V_c_* = 2.6 m/s (red arrows represent the direction of smoke movement).

As we know, with an increase in the cross-wind velocity, the smoke recirculation behavior will be much more obvious inside the canyon. Specifically, the most representative smoke dispersion behavior in street canyons (W = 18 m and H from 9–36 m) under critical recirculation velocity conditions is depicted in Figure 5. We can see that, generally, the smoke dispersion can be categorized into two different regimes:

(1) When the height of the building is greater than 9 m and less than 18 m (i.e., the canyon aspect ratio is 0.5 ≤ *n* ≤ 1, see Figure 5a), the smoke first rises due to buoyancy (slightly tilted toward the windward building), then moves downward under the action of ambient wind and finally sinks to the floor. After that, the entrainment from the fire source will drag the smoke, moving to the fire and rising up again, and thereby smoke recirculation will take place. Such a phenomenon is similar to previous research [20], reflecting a “fully recirculation stage” until the building height reaches H = 18 m, as seen in Figure 5b.

(2) When the building height is greater than 18 m (i.e., the canyon aspect ratio 1 < *n* ≤ 2, see Figure 5c), the smoke will tilt directly toward the leeward building, but the sinking of the smoke at the building facade is different. As seen in the figure, the smoke cannot reach the floor as in the “fully recirculation stage” but is instead recirculated in the middle-upper part inside the street canyon, which we suggest is a “semi recirculation stage”. The main reason is that the mechanical equilibrium condition can be reached for a higher leeward building, so the smoke sinking stops and then rises again due to buoyancy. 

**Figure 6:**
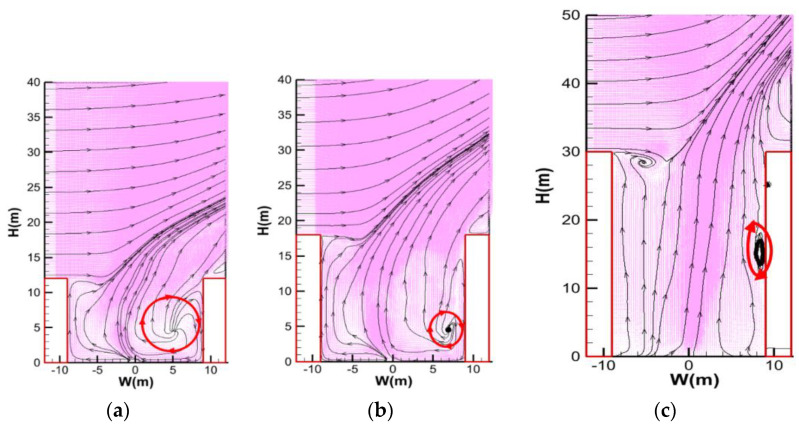
Flow field pattern at Y = 0 m under critical recirculation velocity at 200−400 s: (**a**) H = 12 m, *V_c_* = 3.2 m/s; (**b**) H = 18 m, *V_c_* = 2.8 m/s; and (**c**) H = 30 m, *V_c_* = 2.4 m/s (red circles highlight the vortex position and relative impact on the smoke recirculation).

The typical flow patterns (200–400 s) for the above conditions are depicted in Figure 6. It is seen that for a relatively low building height (i.e., H ≤ 18 m), there is an obvious clockwise vortex in the vicinity of the leeward building, which can slightly push the fire plumes tilted toward the windward building. However, with increasing building height, this vortex disappears, but a relatively weaker vortex close to the wall appears at a higher position near the leeward building, showing good evidence for the smoke “semi recirculation stage”. Moreover, since the buoyancy will become weaker at a higher position, a small clockwise vortex is presented at the top of the windward building, but the smoke will not be dispersed in the street canyon.

### 3.2. The Critical Smoke Recirculation Velocity for Different Street Canyon Conditions

The smoke recirculation behavior inside the street canyon was visually observed according to previous research. The minimum cross-wind velocity that causes smoke recirculation is known as the “critical recirculation velocity” and is expressed as $V_{c}$. It should be noted that even though the smoke can hardly reach the floor in the “semi recirculation stage”, the wind velocity at which the smoke starts to sink down on the leeward building is considered to be critical in this work.

**Figure 7:**
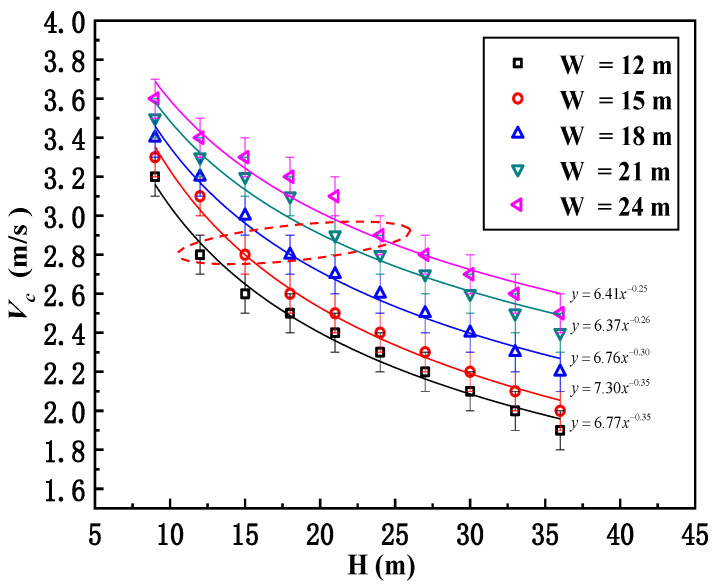
Relationship between critical recirculation velocity and building height for different street canyon widths (the red dashed ellipse marks the ideal street canyon with an aspect ratio of *n* = 1, and the critical recirculation velocity is shown to be almost constant at around 2.8–2.9 m/s).

The critical smoke recirculation velocity for different street canyon conditions is presented in Figure 7. In general, we can see that the critical recirculation velocity is decreased with increasing building height for a given street width. On the contrary, when the canyon width is broadened, the critical recirculation velocity will increase. As we discussed before, the smoke recirculation behavior will reflect the competition between inertia force and thermal buoyancy. Relative to the building height, the thermal buoyancy is weakened at a higher elevation, but the inertia force remains constant, so the smoke recirculation is easier to observe when the building is higher. However, since the smoke traveling distance from the fire source to the leeward building is increased for a wider street, the smoke dispersion to the surroundings becomes easier, and hence, smoke recirculation is more unlikely to take place. The decrease in the critical velocity for different street widths could be effectively proposed through a series of simple equations, such as $V_{c}=\alpha H^{\beta }$, displayed in the right corner of Figure 7. It is seen that the *β* coefficients are all close to −0.3, which is consistent with previous investigations by Hu [31], where $V_{c}\propto {\dot{Q}}^{1/3}H^{-1/3}$. In the meantime, for an ideal street canyon with an aspect ratio of *n* = 1, the critical recirculation velocity is shown to be almost constant at around 2.8–2.9 m/s (see the ellipse in Figure 7); hence, the average critical recirculation velocity for ideal street canyons is suggested to be 2.84 m/s.

Based on the above analysis, we suggest the aspect ratio of the street canyon (i.e., *n* = H/W) to be a main factor determining the smoke recirculation behavior. The relationship between the critical recirculation velocity and the street canyon aspect ratio is further discussed in the next section.

### 3.3. Correlation of the Smoke Critical Recirculation Velocity for Different Street Canyon Aspect Ratio Conditions

**Figure 8:**
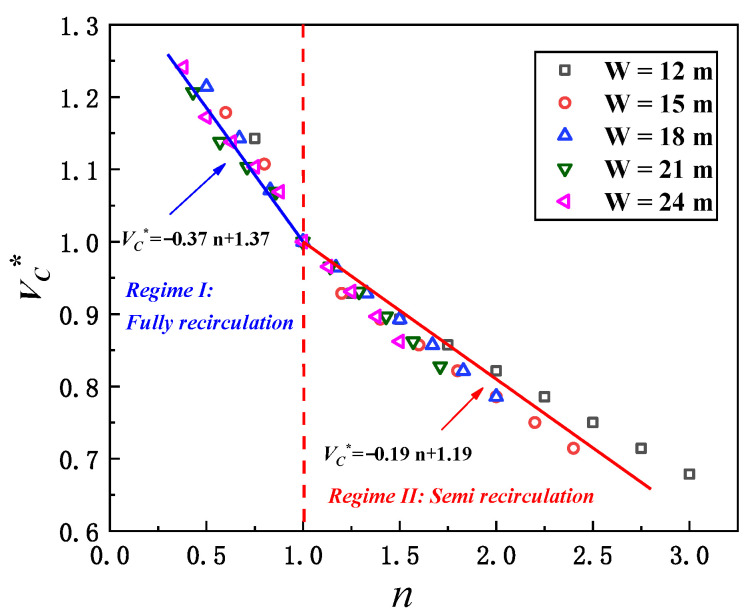
Relationship between dimensionless critical recirculation velocity and aspect ratio under different building separation distances (the transition of smoke “fully recirculation” to “semi recirculation” will take place when the aspect ratio of the street canyon is *n* = 1).

Referring to the critical recirculation velocity for an ideal street canyon, we employed a dimensionless critical recirculation velocity (V_c_*) regarding the critical recirculation velocity for various aspect ratio conditions:

(2) $$V_{c}^{*}=V_{c}/V_{c,0}$$

in which *V_c_* is the critical recirculation velocity in each case, and *V_c_*_,0_ is the critical recirculation velocity for an ideal street canyon with an aspect ratio of *n* = H/W = 1. Thus, Figure 8 shows the relationship between the dimensionless critical recirculation velocity (V_c_*) and the aspect ratio of the street canyon (*n*). It is clear that with the increasing aspect ratio, the dimensionless critical velocity decreases as the buoyancy becomes weaker and inertial force dominates the smoke recirculation. As we stated before, the smoke will be fully recirculated inside the canyon for *n* ≤ 1 cases, but as it becomes semi-recirculated for *n* > 1 cases, we suggest a piecewise function with a distinguishing point at *n* = 1 to be used here. From Figure 8, the data can be well correlated by linear fitting in each stage:

(3) $$V_{c}{}^{*}=-0.37\;n+1.37\;\;\;for\;n\leq 1$$

(4) $$V_{c}{}^{*}=-0.19\;n+1.19\;\;\;for\;n>1$$

It is noted here that when the aspect ratio *n* = 1, the dimensionless critical recirculation velocity V_c_* should also be 1, even though it might not be the best fit. Considering that the average *V_c_*_,0_ is 2.84 m/s for all aspect ratio *n* = 1 cases, the correlation of the smoke critical recirculation velocity with the street canyon aspect ratio condition can be well achieved as:

(5) $$V_{c}=3.89-1.05\;n\;\;\left(m/s\right)\;\;\;for\;n\leq 1$$

(6) $$V_{c}=3.38-0.54\;n\;\;\left(m/s\right)\;\;\;\;for\;n>1$$

**Figure 9:**
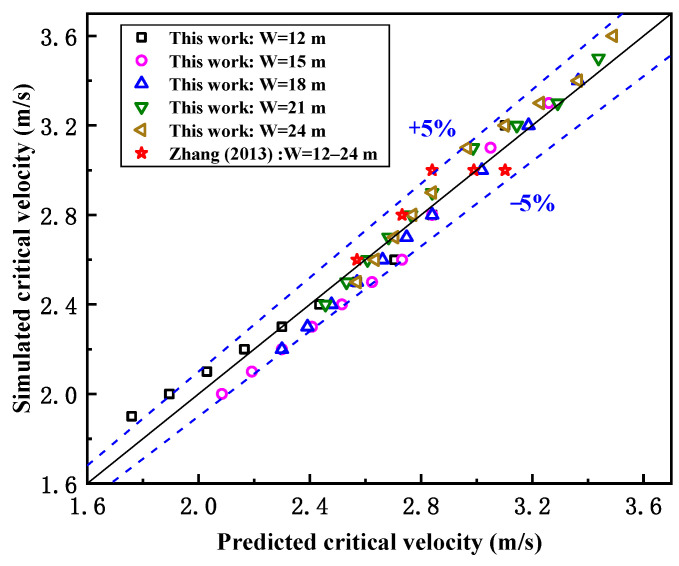
Relative error between predicted value and simulation value in this paper and Zhang’s study (the relative errors of the results are mostly within ±5%).

Figure 9 shows the comparison of the predicted and simulated results of the critical wind velocity for smoke recirculation from street canyon fires with different aspect ratio conditions, and Zhang’s work is also included [30]. In general, the relative errors of the results are mostly within ±5%, and the results of Zhang’s study are very close to the predicted values in this paper. Here, we should note that the above model would not be appropriate when the building height is too low or the street is too narrow due to the fact that the flow would become much more complex in lower places near the ground, while the evolution of the fire plume is strongly dependent on the exterior building wall boundary conditions.

## 4. Conclusions

This paper investigates the smoke recirculation behavior inside a street canyon with different aspect ratio conditions. The smoke dispersion behavior and flow pattern for different groups are presented and compared, and the transition of smoke recirculation inside the street canyon is discovered. The relationship between the critical smoke recirculation velocity and the aspect ratio of the street canyon is further discussed. Major findings include:

1. The smoke recirculation behavior inside the street canyon can be divided into two different regimes for different aspect ratio (*n* = H/W) conditions. The transition of smoke from the “fully recirculation stage” (smoke recirculation in the entire canyon) to the “semi recirculation stage” (smoke recirculation in the middle-upper part of the canyon) will take place when the aspect ratio of the street canyon is *n* = 1.
2. For street canyons with higher buildings and narrower streets, the critical velocity for the presence of smoke recirculation is found to be lower; however, this critical velocity will increase with decreasing building height and increasing street width. The critical smoke recirculation velocity is found to be almost constant at 2.84 m/s when the aspect ratio of the street canyon is *n* = 1.
3. A piecewise function with a distinguishing point at *n* = 1 is proposed for the normalized critical velocity V_c_* (i.e., the critical velocity *V_c_* upon the standard critical velocity for an ideal street canyon *V_c,_*_0_). Within the “fully recirculation stage” and “semi recirculation stage”, the critical velocity decreases linearly with the increasing street canyon aspect ratio.

The present study provides a simple way to predict the critical recirculation velocity in a street canyon, which is beneficial for a better understanding of the flow behavior. From the results, it is found that if the aspect ratio is greater than 1, the risk of smoke recirculation inside the street canyon will be strongly enhanced, imposing much more potential hazards on the health of residents and pedestrians within the street canyon. Still, we should note that the results for the variation in the critical recirculation velocity with the aspect ratio should be limited to a small range of street canyon models, as this work shows. We suggest that some more relevant factors, such as adjacent street canyon groups or structures, asymmetric street canyons (i.e., different building heights on windward and leeward sides) and the ambient wind profile effect, need to be further addressed in the near future.

## Figures and Tables

**Table 1:** Simulation scenarios.

Test No.	Street Canyon Configurations (Length: L = 40 m)										
	W	H									
**1–10**	**12 m**	**9 m**	**12 m**	**15 m**	**18 m**	**21 m**	**24 m**	**27 m**	**30 m**	**33 m**	**36 m**
	*n* = H/W	0.75	1.00	1.25	1.50	1.75	2.00	2.25	2.50	2.75	3.00
**11–20**	**15 m**	**9 m**	**12 m**	**15 m**	**18 m**	**21 m**	**24 m**	**27 m**	**30 m**	**33 m**	**36 m**
	*n* = H/W	0.60	0.80	1.00	1.20	1.40	1.60	1.80	2.00	2.20	2.40
**21–30**	**18 m**	**9 m**	**12 m**	**15 m**	**18 m**	**21 m**	**24 m**	**27 m**	**30 m**	**33 m**	**36 m**
	*n* = H/W	0.50	0.67	0.83	1.00	1.17	1.33	1.50	1.67	1.83	2.00
**31–40**	**21 m**	**9 m**	**12 m**	**15 m**	**18 m**	**21 m**	**24 m**	**27 m**	**30 m**	**33 m**	**36 m**
	*n* = H/W	0.43	0.57	0.71	0.86	1.00	1.14	1.29	1.43	1.57	1.71
**41–50**	**24 m**	**9 m**	**12 m**	**15 m**	**18 m**	**21 m**	**24 m**	**27 m**	**30 m**	**33 m**	**36 m**
	*n* = H/W	0.38	0.50	0.63	0.75	0.88	1.00	1.13	1.25	1.38	1.50
**Global condition**		**HRR:** 5 MW; **Ambient:** 20 °C; **Wind velocity:** from 1.9–3.6 m/s, with intervals by 0.1 m/s									

## Data Availability

The datasets used and/or analyzed during the current study are available from the corresponding author on reasonable request.
